# Individual, family and offence characteristics of high risk childhood offenders: comparing non-offending, one-time offending and re-offending Dutch-Moroccan migrant children in the Netherlands

**DOI:** 10.1186/1753-2000-5-33

**Published:** 2011-10-20

**Authors:** Carmen H Paalman, Lieke van Domburgh, Gonneke WJM Stevens, Theo AH Doreleijers

**Affiliations:** 1 VU University Medical Centre, Department of Child and Adolescent Psychiatry, Amsterdam, the Netherlands; 2 LSG-Rentray, Zutphen, the Netherlands; 3 Utrecht University, Interdisciplinary Social Science, Faculty of Social Sciences, Utrecht, the Netherlands; 4 Leiden University, Faculty of Law, Leiden, the Netherlands

**Keywords:** childhood onset delinquency, childhood onset offending, migrant, ethnicity, risk factors

## Abstract

**Background:**

Childhood offenders are at an increased risk for developing mental health, social and educational problems later in life. An early onset of offending is a strong predictor for future persistent offending. Childhood offenders from ethnic minority groups are a vulnerable at-risk group. However, up until now, no studies have focused on them.

**Aims:**

To investigate which risk factors are associated with (re-)offending of childhood offenders from an ethnic minority.

**Method:**

Dutch-Moroccan boys, who were registered by the police in the year 2006-2007, and their parents as well as a control group (n = 40) were interviewed regarding their individual and family characteristics. Two years later a follow-up analysis of police data was conducted to identify one-time offenders (n = 65) and re-offenders (n = 35).

**Results:**

All groups, including the controls, showed substantial problems. Single parenthood (OR 6.0) and financial problems (OR 3.9) distinguished one-time offenders from controls. Reading problems (OR 3.8), having an older brother (OR 5.5) and a parent having Dutch friends (OR 4.3) distinguished re-offenders from one-time offenders. First offence characteristics were not predictive for re-offending. The control group reported high levels of emotional problems (33.3%). Parents reported not needing help for their children but half of the re-offender's families were known to the Child Welfare Agency, mostly in a juridical framework.

**Conclusion:**

The Moroccan subgroup of childhood offenders has substantial problems that might hamper healthy development. Interventions should focus on reaching these families tailored to their needs and expectations using a multi-system approach.

## Background

Previous research has established a strong relation between an early onset of delinquent behaviour and future persistent offending [1-5]. Childhood offenders, i.e. children who display delinquent behaviour prior to the age of twelve^1^, are two to three times more likely to become serious and persistent offenders than those with a later onset [4,6,7]. In addition, these children have an increased risk of developing mental health, social and educational problems during their lives [7-9]. Most research on childhood offending is based on general population studies in which childhood offenders have been analyzed as a homogeneous group [9,10]. However, not all children have a similar risk of starting offending in childhood and not all childhood offenders are as likely to re-offend. According to self-reports approximately 15% of all children display a stable pattern of antisocial and offending behaviour during childhood, of whom only half will persist in serious offending during adolescence. Children living in disadvantaged neighbourhoods are known to have an elevated risk of starting delinquent behaviour as compared to children from more affluent neighbourhoods [7,11]. Among children from disadvantaged neighbourhoods, children from ethnic minorities are at an even higher risk of becoming childhood offenders when compared to Dutch children from comparable neighbourhoods [10]. Despite this risk most children from ethnic minorities living in disadvantaged neighbourhoods do not become childhood offenders. Moreover, those who do will not necessarily persist in delinquent behaviour. In order to appropriately target interventions and address the relevant risk factors, it is essential to gain insight into which risk factors are associated with offending and re-offending. Therefore, this study focuses on risk factors that may distinguish non-offenders from one-time offenders and re-offenders in a high-risk group of ethnic minority boys from disadvantaged neighbourhoods in the Netherlands.

Officially registered offending is in particular a strong predictor for a persistent pattern of delinquency [12]. Nevertheless, most knowledge of childhood offenders is currently based on self-report studies in the general population. Risk factors found for childhood offending are for instance: individual risk factors like mental health problems and problems at school, family risk factors like large families, financial problems, parental delinquency and other parenting problems, and environmental risk factors like living in a disadvantaged neighbourhood and affiliation with delinquent peers. Studies focusing on risk factors of officially registered childhood offenders remain scarce and studies examining the risk factors of registered *re*-offending childhood offenders are even scarcer [13-17]. Furthermore, studies are inconclusive regarding characteristics differentiating one-time offenders from re-offenders. For instance, whilst some found that persisters are more likely to come from dysfunctional families living in disadvantaged neighbourhoods compared to one-time offenders [e.g.,[14,16]], others found no differences in individual, family or neighbourhood characteristics between one-time and re-offending children [9,13]. Additionally, some have stressed the predictive value of violent offences, whereas others found that less serious offences are equally predictive of a persistent pattern of offending [13,18,19]. Nevertheless, most researchers agree on a high probability of an early police encounter for boys from ethnic minorities from disadvantaged neighbourhoods [10,12,20,21].

However, ethnicity alone is uninformative about *which *characteristics put these children at an increased risk, as it is not known whether risk factors found for offending in general populations also hold for childhood offenders from ethnic minorities. More importantly, it is unclear which risk factors differentiate one-time offenders from re-offenders among childhood offenders from ethnic minorities. Van Domburgh et al. [10] found that among non-Western children from disadvantaged neighbourhoods, a combination of individual, peer and parental problems differentiated the level of childhood offending. However, these results can not be generalized since this study included all non-Western children, while these children in fact comprise a heterogeneous group.

Certain minorities tend to be over-represented in the national justice systems and in institutions for delinquent youth. Like Algerians in France, Turks in Germany and West-Indians in England, Moroccans are over-represented in police and justice systems in the Netherlands [22-24]. Moroccan immigrants belong to one of the largest migrant groups in the Netherlands. Currently, two percent of the Dutch population is of Moroccan origin. Migration began in the 1960s when Moroccan man were recruited for working in the Dutch labour market. Nowadays, about 40% of the Moroccan immigrants are born in the Netherlands. Dutch police records show that Moroccan juveniles, in comparison to both native Dutch and other ethnic minority groups, are over-represented in the population of juvenile delinquents and in justice youth care [25-27]. There are many reasons for this over-representation, including racial discrimination, selective arrest and intake in the justice system and a high exposure to risk factors associated with delinquency [7]. For instance, Moroccans communities in the Netherlands face social-economic disadvantaged like poverty, unemployment and poor housing conditions [28]. Furthermore, certain individual risk factors, like behavioural problems, may exert a relatively strong influence on childhood offenders with a Moroccan background (further called Dutch-Moroccans) as these problems tend to remain untreated among Dutch-Moroccan youth and may escalate into delinquent behaviour later [29-31]. As a result, mental health care for Dutch-Moroccan youth is often characterized by a juridical framework [32]. Moreover, Dutch-Moroccan children have language problems from the beginning of elementary school onwards, which is strongly associated with educational problems and dropping out later on [33]. In addition to these somewhat general risk factors, specific risks among ethnic minorities like acculturation problems have been related to delinquency [34-36]. Acculturation is the way in which people relate to their ethnic and host culture. It is assumed that a strong orientation to both ethnic and host cultures gives the best quality of life for children and therefore leads to the lowest risk of delinquent behaviour [37]. In contrast, using Merton's strain theory [38] migrants who are strongly orientated towards the host society are at an increased risk of delinquent behaviour because of discrepancies between pursued goals and possibilities to achieve those goals. In addition, instead of integrating into the host's middle class, migrants more often unintentional integrate into the host's 'underclass' where delinquency is more prevalent. This may also increase delinquency in those integrated migrants [39].

In summary, there are many risk factors associated with offending present in Dutch-Moroccans in the Netherlands. However, it is unclear which risk factors differentiate between non-offending, one-time offending and re-offending in a high-risk group of Dutch-Moroccan boys. Insight into these risk factors is of great importance in order to tailor interventions while maximizing efficiency. Therefore the aim of this study was to investigate which individual, family and acculturation risk factors differentiate non-delinquent, one-time offending and re-offending Dutch-Moroccan boys. In addition, offence characteristics between one-time offenders and re-offenders are compared.

Given the high-risk profile of Dutch-Moroccan boys in the Netherlands, we expected most participants in our study to have individual and family characteristics that are generally acknowledged as risk factors for offending. Overall, we expected these risk factors to be most prevalent in re-offenders. Due to their low attendance at voluntary mental health care facilities and the strong association between behavioural problems and delinquent behaviour, we expected re-offenders to have more behavioural problems and to have received more mental health care within the juridical framework. In addition, we expected offenders and re-offenders to be more oriented towards Dutch society compared to the controls.

This present study is to our knowledge the first study that focuses on a high-risk subgroup of childhood one-time offenders and re-offenders from a single ethnic minority group. Moreover, instead of self-reported delinquency, we used police registration to define one-time offenders and re-offenders and compared these boys with a matched group of non-delinquent Dutch-Moroccan boys. Finally, whereas most studies rely on either self-reports or police registrations, this study made use of multiple sources: official police registrations, child and parent reports and information from the Child Welfare Agency.

## Methods

### Participants and procedure

Participants in the study were 97 male childhood offenders who were registered by the police before the age of twelve (mean age 10.68 ± 1.48). All participants were of Moroccan origin, lived in Amsterdam and were registered by the police in 2006-2007. Seventy-two percent of the boys who were requested to participate took part in the study. Non-responders did not differ from responders in age at first arrest, neighbourhood SES and type of offence. Permission to approach the eligible participants was obtained by the city authority and the study was approved by the Dutch Ministry of Justice. Trained, female Moroccan researchers gave oral and written information in Dutch and Moroccan Arabic about the study and obtained written informed consent from both the child and a parent. Confidentiality of their responses was assured and data was archived anonymously. Participating children received a small gift and parents received a gift voucher. As indicated by family income, employment and educational level, all participants resided in low to very low SES neighbourhoods [40]. In addition, a control group of 43 Dutch-Moroccan boys residing in the same neighbourhoods without registered police contacts before the age of twelve was composed (mean age 9.71 ± 1.40). Recruitment of these children took place at elementary schools in matched neighbourhoods of the offenders. Schools sent information about the study to the parents in Dutch and Moroccan Arabic. After permission from the parent(s), an appointment was made for an interview.

Two years after the initial data-collection, police data were collected to identify re-offenders. Re-registration for delinquent behaviour within two years of the initial registration was defined as re-offending. Boys without new registrations within two years of the initial registration were called one-time offenders. The control group comprised children without registered police contact before the age of twelve. One boy of the original control group was found to have a police contact before age twelve in the two year follow-up time and was therefore re-assigned to the one-time offender group. In addition, two boys in the original control group had two police contacts in the two-year period and were therefore re-assigned to the re-offender group. This resulted in 35 re-offenders and 65 one-time offenders and a control group of 40 non-offenders.

### Measurements

#### Individual characteristics

Behavioural and emotional problems of the boys were measured using the parent and child reports of the Strengths and Difficulties Questionnaire (SDQ) [41,42]. The SDQ is a 25-item behavioural screening questionnaire which has been translated into more than 40 languages http://www.sdqinfo.org and was validated in several cultures, including Arabic [43]. For this study, the following subscales were used: emotional problems, behavioural problems, hyperactivity and peer problems. The internal consistency is generally good for both parent and child reports (α = .81 and α = .72) [41]. Scores can be divided into normal, borderline and clinical range. In this study, cut-offs were based on clinical scores which normally includes about 10% of the scores.

Reading problems were assessed using the 1-Minute Reading Task, in which children are requested to read as many words correctly as possible within a time frame of one minute [44]. A boy was considered to have reading problems when he was more than one year behind the level considered appropriate for children of his age and school year, taking repeated years into account. Information on repeated years (from elementary school) was obtained through self-report.

#### Delinquent Peers

Affiliation with delinquent peers was assessed with a four-point item derived from the Social and Health Assessment (SAHA): how many of your friends have had police contact (none = 0 to most/all = 3)? The SAHA is an assessment package combining various instruments on child behaviour, health and development and has been used for youth population studies in various countries [45,46]. The SAHA includes both new scales and existing, validated scales. The original version was developed by Weissberg et al. [47] and has been adjusted for specific population over the years [e.g. [48,49]].

#### Family characteristics

Parent reports on general demographics were used to determine family size, country of birth and financial problems.

Arrest rates and domestic violence were obtained from police registrations. If violence in the family or home sphere was reported in any police record, this was used as an indication of domestic violence.

To assess low positive parenting, the affection and discipline scales of the Nijmegen Rearing Questionnaire were used [50]. This questionnaire was developed in 1993 to measure child-rearing processes of parental support and control in the context of a national survey on parenting in the Netherlands. The affection and discipline scales assess the extent to which the parents show feelings of positive affection toward their sons and measure different means of punishment and discipline that parents may use. Parents were asked to indicate their agreement or disagreement on a five-point scale (0 = completely disagree to 4 = completely agree). Internal consistency was good (both scales α = .70). Also, the son's perspective on positive parenting was measured using the SAHA. The 11 items on the child's perception of parental involvement and warmth showed an internal consistency of α = .68. Parental control was measured by a five-item questionnaire [51] on a four-point scale (0 = nothing to 3 = everything). Parents were asked, for example, how much they know about their son's friends or how their son spends his money. The son's perspective on parental control was measured by means of an eight-item subscale of the SAHA. This instrument measures the child's perception of parental control by items such as "My mother wants to know if I have done my homework" and "My mother wants to know with whom I hang around". For both parent and child reports on positive parenting and parenting control, the lowest third of scores was used as the cut-off for low positive parenting and low parental control.

#### Acculturation

An adapted version of the Psychological Acculturation Scale (PAS) was used to measure both child's and parent's sense of belonging and being emotionally attached to Dutch (D-PAS) and/or Moroccan (M-PAS) society [52]. The PAS was originally developed to assess emotional attachment to, belonging within, and understanding of the Anglo American and Latino/Hispanic cultures [53]. Stevens et al. adapted items to Dutch and Moroccan culture and translated the instrument into Dutch and Moroccan-Arabic. Independent back translation into English were performed to check the accuracy of the translation [52]. Items were rated on a five-point Likert scale and included for instance 'Dutch people understand me' and 'Moroccan people understand me' and 'I feel proud of Dutch culture' and 'I feel proud of Moroccan culture'. Internal consistency was good for parent reports with α = .82 for both the D-PAS and the M-PAS. For the boys, internal consistency was also good with α = .78 for the D-PAS and α = .86 for the M-PAS. Mean item scores on both D-PAS and M-PAS were used to compare groups.

In addition, both parent and child were asked whether they considered themselves Dutch and whether they had one or more Dutch friends.

#### Offence characteristics

Offending was defined as registered behaviour that could be prosecuted or fined if displayed at the age of twelve or older (the age of criminal responsibility in the Netherlands). Irrespective of age, local police should register all individuals that display, or are suspected of, delinquent behaviour. Next to registrations of those who were caught by the police while offending, we also included offending behaviour reported by third parties, such as schools reports on thefts that were dealt with by the school or an issued prohibition by a swimming pool. Unsuccessful attempts at offending, and highly suspicious behaviour registered by the police, such as trying to unlock bikes with tools or trying to enter private property, were also included. Re-offending was defined if the police registered a boy for an offence within two years of the first registered offence. Giving the focus on childhood delinquents in this study, we choose a follow-up period of two years. That way, most children did not enter middle adolescence yet, a period in which delinquent behaviour increases [2]. In addition, previous studies showed that the majority who will re-offend, will do so in the two years following their first arrest [54].

Type of offending was classified into violence (both verbal and physical), theft, property damage and mischief. In addition, seriousness of offending was determined by using the Seriousness of Early Police Registration (SEPR) classification [55,56]. The SEPR distinguishes five levels of seriousness for offending: Level 1: Minor delinquency at home, minor verbal aggression and rule breaking behaviour. Level 2: Minor delinquency outside the home, e.g., shoplifting and minor vandalism. Level 3: Moderate delinquency, e.g., fighting without bodily harm, vandalism and theft. Level 4: Serious delinquency, e.g., breaking and entering, serious arson and vehicle theft. Level 5: Very serious delinquency, e.g., sex offences, aggravated assault and robbery.

Two independent researchers rated seriousness and type of offending. In case of inconsistencies, a consensus meeting resulted in the scores finally used.

#### Health care consumption

Parents were asked by means of a structured questionnaire to provide information about health care consumption related to their son's behaviour. For example, parents were asked whether they had received help for their son's behaviour or whether they were in need of help for their son's behaviour. In addition the Child Welfare Agency (Bureau Jeugdzorg) database was searched to find out whether the boy was known to the agency, and whether this contact was voluntary or obligatory. Due to privacy reasons, the Child Welfare Agency could only provide us the data on the group level (controls, one-time offenders and re-offenders).

#### Social desirability

Because of assumptions about high socially desirable responses among ethnic minorities, parents answered the ten items of the Marlowe-Crown Social Desirability Scale to assess social desirability [57]. According to this scale all parents indeed answered socially desirable (range 1-10: controls 8.97 ± 1.44; one-time offenders 9.45 ± 1.04; re-offenders 9.33 ± 1.07). The children were presented with ten items from the Social Fear Scale for children which has Dutch norms [58]. According to this scale, 3.1% of the controls answered socially desirable, 21.6% of the one-time offenders and 17.2% of the re-offenders.

### Statistical analysis

For all analyses, SPSS version 17 was used. For better interpretation, most variables were dichotomized and described using percentages. The remaining continuous variables were described using means. Initially, group comparisons were conducted using univariate logistic regressions, with confidence intervals of 95%. We performed separate analyses to investigate differences between the three groups. In order to do so, dependent variables were 1. control versus one-time offenders, 2. one-time offenders versus re-offenders and 3. re-offenders versus controls. Next, significant characteristics identified in the separate univariate analyses were entered into a backward multiple logistic regression analysis. One by one, the variable with the lowest Wald, was removed from the analyses until only significant variables remained in the model. Because of the relatively small sample size, a maximum of five variables with the highest odds ratios from the univariate analyses could be entered reliably. Chi-square testing of the difference between the two log-likelihood ratios determined the best model with unique predictors. Multicollinearity proved not to be an issue.

## Results

Table 1 shows prevalence rates and odds ratios (OR) for the individual, peer, family and acculturation characteristics of the controls, the one-time offenders and the re-offenders. In general, all groups showed substantial problems on both individual and family domains, including reading problems, financial problems, family member arrest and domestic violence.

**Table 1:** Descriptives and odds ratios of characteristics of controls, one-time offenders and re-offenders

				**Group comparisons (odds ratio (95%CI))**		
	
	**Control** **(n = 40)** **%**	**One-time offenders** **(n = 65)** **%**	**Re-offenders** **(n = 35)** **%**	**Control vs one-time offenders**	**One-time offenders vs** **re-offenders**	**Re-offenders vs control**
	
**Individual characteristics**						
SDQ (child/*parent report*)						
Emotional problem	33.3/*5.0*	14.3/*9.2*	9.4/*5.7*	33 (.12-.94)*		.21 (.10-.83)*
Behavioural problems	15.2/*12.8*	12.5/*18.8*	9.4/*5.7*		*26 (.06-1.2)^†^*	
Hyperactivity	0.0/*5.0*	3.6/*7.7*	3.1/*8.6*			
Poor relationship with peers	18.2/*15*	17.9/*23.1*	6.3/*20.0*			
Repeated school year	22.6	16.4	37.5		3.1 (1.1-8.4)*	
Reading problems	41.4	56.9	81.3		3.3 (1.2-9.4)*	6.1 (1.9-19.5)**
**Affiliation delinquent peers**	15.2	21.4	37.5			3.4 (1.0-11.1)*
**Family characteristics**						
>3 children at home	57.5	55.4	74.3		2.3 (1.0-5.7)^†^	
Older brother	51.5	62.5	87.5		4.2 (1.3-13.7)*	6.6 (1.9-23.0)**
Single parent	5.0	33.8	22.9			5.6 (1.1-28.6)*
Financial problems	42.5	72.3	69.7	9.7(2.1-44.1)***		3.1 (1.2-8.2)*
(Any) household member arrest	27.5	41.5	62.9	3.5 (1.5-8.1)**	2.4 (1.0-5.5)*	4.5 (1.7-11.8)**
Arrested brother	15.0	32.3	48.6			5.4 (1.8-16.0)**
Arrested father	10.0	13.8	14.3	2.7 (1.0-7.4)^†^		
# total arrests household ¹	0.7(1.4)	1.9(4.4)	3.9(5.8)^a**b† ^			
Low positive parenting						
child report	53.1	28.6	28.1			.35 (.12-.97)*
parent report	37.5	35.4	25.7	.35 (.14-.87)*		
Low parenting control						
child report	50.0	33.9	31.3			
parent report	20.0	10.9	20.0			
Domestic violence	35.0	36.9	37.1			
**Acculturation characteristics**						
Both parents born in Morocco	92.5	76.4	86.7	.26 (.07-1.0)*		
Orientation Dutch society ¹						
child (range 1-5)	3.48(.79)^c^***	4.15(.83)	4.21(.64)			
parent (range 1-5)	3.91(.76)	3.86(.92)	4.30(.78)^d^*			
Orientation Moroccan society ¹						
child (range 1-5)	4.08(.82)^c^*	4.52(.75)	4.53(.53)			
parent (range 1-5)	4.66(.44)	4.55(.64)	4.70(.67)			
Considers Dutch child	21.2	29.8	40.0			
Considers Dutch parent	20.0	12.7	28.6		2.8 (1.0-7.8)^†^	
Dutch friends child	78.8	66.7	76.7			
Dutch friends parent	22.5	33.3	57.1		2.6 (1.1-6.1)*	5.3 (1.8-15.5)**

† p < .1, * p < .05, ** p < .01, *** p < .001. Due to rounding error some of the CI include 1.0. ^¹ ^= mean (sd), ^a ^= difference between re- offenders and control group, ^b ^= approaching significant differences between re-offenders and one-time offenders, **^c ^= **difference between controls and re-offenders/one-time offenders. **^d ^= **difference between re-offenders and one-time offenders

As for the individual characteristics, there were hardly any differences on reported problems in psychosocial functioning between the groups, although a considerably smaller percentage of the one-time offenders and the re-offenders scored in the clinical range on emotional problems (OR .33 and OR .21 respectively) compared to the controls. These emotional problems, as measured with the SDQ, were the only individual characteristic that distinguished one-time offenders from the controls. In contrast, clear differences between re-offenders and one-time offenders were found regarding problems at school. Re-offenders more often faced reading problems compared to one-time offenders (OR 3.3) as well as compared to the controls (OR 6.1). Moreover, re-offenders repeated a school year more often than one-time offenders (OR 3.1). Finally, re-offenders more often had delinquent friends compared to the controls (OR 3.4).

In conclusion, with the exception of fewer emotional problems, we could not find individual characteristics that distinguished one-time offenders from the controls. However, re-offenders were distinguished from the other groups by problems at school and delinquent friends as compared to the controls.

Regarding the family domain, financial problems, domestic violence and an arrest of at least one family member were prevalent in all three groups. Nevertheless, large differences showed up when comparing the three groups. Compared to the controls, the one-time offenders were more likely to grow up in a single parent household (OR 9.7), more often faced financial problems (OR 3.5) and more frequently had a brother arrested (OR 2.7). On the other hand, one-time offenders reported low positive parenting less often (OR .35). Remarkably, other characteristics distinguished re-offenders from one-time offenders. Re-offenders more often lived in large families (OR 2.3) and more often had an older brother (OR 4.2) compared to the one-time offenders. In addition two-thirds of the re-offenders had an arrested family member as compared to over forty percent of the one-time offenders (OR 2.4).

Concluding, family characteristics differed between controls and one-time offenders but also between one-time offenders and re-offenders. Re-offenders demonstrated the highest level of problems concerning family characteristics.

As for acculturation, table 1 shows that the parents of one-time offenders were less often born in Morocco compared to the controls (OR .26). One-time offenders were more oriented towards both Dutch and Moroccan societies compared to the controls. Furthermore, parents from re-offenders were more oriented towards the Dutch society compared to parents from one-time offenders. Re-offenders were also more oriented towards both Dutch and Moroccan societies compared to the controls. Furthermore, parents of re-offenders most often had Dutch friends compared to the controls (OR 5.3) and one-time offenders (OR 2.6).

Concluding, re-offenders and their parents seem mostly oriented towards the Dutch society, while controls and their parents seem least oriented towards the Dutch society.

In table 2 first offence characteristics of the one-time offenders and re-offenders are compared in order to study whether these characteristics were predictive of re-offending. Results showed no differences in type of first offence and seriousness of first offence between one-time offenders and re-offenders. Re-offenders were slightly older at their first arrest and less often committed their offence alone as compared to one-time offenders.

**Table 2:** Offence characteristics of childhood one-time offenders and re-offenders

	One-time offenders (n = 65) %	Re-offenders (n = 35) %	sig
**Age onset first offence **mean (sd)	9.9 (1.3)	10.7 (1.4)	t = -2.958, p = .004
**Offence characteristics**			
type of first offence			
theft	21.9	36.4	ns
violence	23.4	27.3	ns
property damage	25.0	18.2	ns
mischief	29.7	15.2	ns
seriousness first offence			
minor	55.7	46.9	ns
moderate	41.0	50.0	ns
serious	3.3	3.1	ns
solo offending	30.6	15.2	χ = 2.738, p = .098

Next, the significant characteristics associated with one-time offending and re-offending, were entered into a regression model to study which characteristics uniquely contributed to both offending and re-offending. Table 3 shows that the unique characteristics associated with one-time offending were within the family domain (single parent: OR 6.0, financial problems: OR 3.9 and low positive parenting: OR .31) and not within the individual domain. The most important characteristics distinguishing re-offenders from one-time offenders were reading problems (OR 3.8), having an older brother (OR 5.5) and a parent having Dutch friends (OR 4.3). When comparing re-offenders to controls, financial problems (OR 7.8), having an older brother (OR 6.1), reading problems (OR 10.6) and the parent having Dutch friends (OR 14.0) remained important characteristics associated with re-offending.

**Table 3:** Multivariate prediction models of offending and re-offending

	B(SE)	Wald	p	Odds (95% CI)
**Controls/one-time offenders**				
Single parent	1.814 (.833)	4.738	.030	6.0 (1.2-31.4)
Financial problems	1.352 (.513)	6.941	.008	3.9 (1.4-10.6)
Low positive parenting(child report)	-1.174 (.513)	5.246	.022	.31 (.11-.84)
Overall model: χ^2 ^19.003(3), p < .001, Nagelkerke R^2 ^.266				

**One-time offenders/re-offenders**				
Reading problems	1.346 (.604)	4.970	.026	3.8 (1.2- 12.5)
Older brother	1.700 (.687)	6.112	.013	5.5 (1.4-21.1)
Parent Dutch friends	1.466 (.539)	7.406	.007	4.3 (1.5-12.4)
Overall model: χ^2 ^18.749(3), p < .001, Nagelkerke R^2 ^.282				

**Re-offenders/controls**				
Reading problems	2.636 (.883)	8.911	.003	14.0 (2.5-78.8)
Older brother	2.444 (.957)	6.522	.011	11.5 (1.8-75.1)
Financial problems	1.777 (.816)	4.745	.029	5.9 (1.2-29.2)
Parent Dutch friends	2.638 (.874)	9.119	.003	14.0 (2.5-77.5)
Overall model: χ^2 ^44.369(4), p < .001, Nagelkerke R^2 ^.616				

Information regarding mental health care was gathered through parent reports and through the Child Welfare Agency. Results in table 4 show that, although half of the re-offenders had received help at some point, none of the parents indicated they were in need of help for their son's behaviour at that moment. In line with this result, over fifty percent of the re-offenders had received mental health care within a juridical framework. Of the one-time offenders about two-thirds of the parents had received help for their son's behaviour. Twenty-five percent of the one-time offenders had received mental health care within a juridical framework, while just over ten percent of the parents indicated they were in need of help for their son's behaviour. In contrast, in the control group there was no discrepancy between parents' need for help for their son's behaviour and their mental health care consumption at that moment. Over one quarter had received help for their son's behaviour at some point, while none received mental health care within a juridical framework specifically.

**Table 4:** Health care consumption of controls, one-time offenders and re-offenders

	Control (n = 40) %	One-time offenders (n = 65) %	Re-offenders (n = 35) %
**Health care parent report**			
Received help for child's behaviour	26.7	35.9	48.5
In need of help for child's behaviour	5.7	12.7	0.0

Known at Child Welfare Agency*	6.8 (n = 3)	35.8 (n = 24)	44.1 (n = 15)
Forensic/compulsory care	0	25	53.3
**In care at time of research**	4.5 (n = 2)	20.9 (n = 14)	14.7 (n = 5)

*based on group information from the Child Welfare Agency

## Discussion

The aim of this study was to investigate which individual, family, acculturation and offence characteristics were associated with offending and re-offending in a high risk sample of Dutch-Moroccan boys residing in disadvantaged neighbourhoods. Regarding individual risk factors our hypothesis was partly confirmed. Problems at school were prevalent in all boys, but re-offenders more often reported having problems at school compared to one-time offenders and controls^2^. Although, re-offenders did not report more mental health problems, as measured by the SDQ, in line with our hypothesis, they received their mental health care more often within a juridical framework. In contrast, the control group more often reported emotional problems compared to the re-offender group. In line with our hypothesis, family risk factors, such as single parenthood, financial problems, family member arrest and domestic violence, were often present regardless of the level of offending^3^. Furthermore and in line with our hypothesis, family risk factors were most prevalent among re-offenders and least present among controls. As expected, re-offenders were most acculturated toward the Dutch society compared to the controls and one-time offenders. Finally, first offence characteristics were not associated with re-offending in this group of childhood offenders. While the main risk factors for offending were within the family domain, risk factors for re-offending were found in the individual domain as well. Most important factors for re-offending were reading problems, having an older brother, financial problems and a parent having Dutch friends.

### Individual characteristics

Contrary to earlier findings on the positive relation between mental health problems and delinquent behaviour in the general population [59-62], the current study found no relation between most mental health problems as measured with the SDQ and offending. Because delinquent behaviour can be considered a symptom of behavioural problems, we expected in particular (re-)offenders to have behavioural problems. In contrast to this expectation, behavioural problems did not differentiate (re-)offenders from controls. However, behavioural problems were measured by means of child and parent reports and despite the fact that behavioural problems were seldom reported by children or their parents, a large percentage of the (re-)offenders was nevertheless known to the Child Welfare Agency. This might reflect a discrepancy between what parents consider problematic behaviour and what others, e.g. police and health care professionals, consider as such. This is in line with previous research stating that Moroccan parents have a lower identification rate of behaviour problems compared to other ethnic groups [63]. Socially desirable answering may also play a role, since all parents in our study scored equally high on socially desirable answering. This also holds for the children; one-time offenders and re-offenders reported high rates of socially desirable answering. It is therefore possible that behavioural problems are under-reported or not recognized in this group.

On the other hand, low reported levels of behavioural problems might truly reflect low rates of these problems. Low levels of mental health problems have been previously reported among adolescent offenders from ethnic minorities [64,65]. Also, a recent study on incarcerated Dutch-Moroccan youths in The Netherlands showed low levels of both internalizing and behavioural problems in these youths [66]. It has been hypothesized that disparities in sentencing procedures may play a role in the police contacts of Dutch-Moroccans with relatively low levels of mental health problems [66]. Furthermore, the boys in our study lived in the most disadvantaged neighbourhoods, characterized by less safety and more police on the streets, elevating the chance of getting caught.

It also may be that not the behaviour itself, but the way the environment (is able to) react to these problems, e.g. due to other stressors, such as parenting or poor role models, that determine who will display delinquent behavior that is registered by the police.

High levels of emotional problems as measured with the SDQ were reported by the controls whereas both one-time offenders and re-offenders did not report such problems. The environment in which the boys in our study reside, i.e. low neighbourhood SES, household arrests, financial problems, has been related to both externalizing behaviour like delinquency [17,67,68] and internalizing problems like depression and anxiety [69-72]. Mediating factors, like parenting style, the child's temperament and cognitive functioning may explain the different developmental pathways to either delinquent behaviour (externalizing) or emotional problems (internalizing). Although not consistently reported, emotional problems have been found protective for delinquent behaviour in some studies [73,74]. Future research could focus on these different developmental pathways and their mediating factors in subgroups of high risk children.

### Family characteristics

The elevated problems in the family domain of one-time offenders and re-offenders are in line with findings from previous studies [2,3,17]. Patterson's Social Interaction Learning model outlines developmental delinquency trajectories for youth. In this model the relation between living in stressful circumstances and the development towards delinquent behaviour highly depends on how well parents are able to maintain positive parenting strategies under these circumstances [75,76]. The more stressful the circumstances are, the harder it is to regulate or act pro-social on signals of deviant behaviour of children. In our study the re-offending group lived in the most stressful circumstances with high levels of single parenthood, high household arrest rates and large families. However, according to the child, positive parenting was higher in the one-time offenders and re-offenders as compared to the controls, although this could not prevent the child's delinquent behaviour. In this study positive parenting was overshadowed by other family characteristics like single parenthood and financial problems. Having an older brother was an important risk factor for re-offending in our study. Moroccan families in the Netherlands are known to have traditional hierarchical family structures in which the (oldest) man is typically head of the family [34]. Being an older brother comes with responsibilities and might prevent delinquent behaviour. In contrast, being a younger brother comes with fewer responsibilities and therefore might be a risk factor for delinquent behaviour. Although there has been research on relations between sibling relations and delinquent behaviour [77-79], these studies primarily focus on Caucasian families. Future research should study these associations within different cultural contexts. However, in our study, a large percentage of the older brothers had been arrested. Especially the re-offending boys have brothers that can be considered as poor role models for their younger brothers.

### Acculturation

Although in this study we only measured a few selected characteristics on acculturation, results indicated a stronger orientation towards Dutch society by the (re-)offenders compared to the controls, as expected. Especially those who are strongly oriented toward Dutch society may be more sensitive to their disadvantaged position. As a result, feelings of frustration may enhance delinquent behaviour [36,38,39]. Our findings are in line with results from a recent study by Veen et al., (2011), who found incarcerated Dutch-Moroccan boys to be more orientated toward Dutch society compared to a control group of non-offending Dutch-Moroccan boys [27].

### Implications

It is clear that the group studied has many risk factors that may hamper the healthy development of a child. Most of these risk factors, such as socio-economic risk factors, have been put forward in the literature and although often prevalent in Dutch-Moroccan boys, they are not unique to this group. Moreover, such risk factors are not unique for childhood delinquency and/or persistence, but are also found to be risk factors for adolescent offending [e.g. [55]]. Meaning that, apparently there are general risk factors for delinquency, irrespective of subgroup, persistency or age of onset. Acculturation characteristics and having an older brother seem specific risk factors for re-offending in this specific group of Dutch-Moroccan boys in the Netherlands. Reading problems may also be considered a problem related to Dutch-Moroccan children. In migrant children there is a strong association between reading difficulties and language problems [80]. This deficit can be made up by investments in pre-school education and focus on language and reading training throughout elementary school in order to help prevent further educational problems. In addition, preventing educational problems creates more opportunities to be part of Dutch society, which in turn may decrease delinquent behaviour.

Given the low levels of behavioural problems according to self-reports, a police encounter may be regarded as an opportunity to screen and, if needed, intervene in families that do not tend to seek help themselves. Complicating factor is the fact that parents of (re-)offenders may not agree they are in need of help. An important challenge for health care agencies is to actually reach these families and to formulate shared goals to prevent further escalation. The high prevalence of family risk factors stresses the importance of a multi-system approach, taking the child, the family and the broader environment into account. Since older brothers were found to be a risk factor for offending and re-offending, it may help to improve the position of the brothers as positive role models, for instance by creating more job and schooling opportunities.

Not only boys displaying delinquent behaviour, but also the controls from comparable disadvantaged neighbourhoods need our attention, considering their stressful social environment and high levels of reported emotional problems. These children are especially hard to reach, since parents might have a lower detection rate of problems and the police do not see these children. Outreaching and culturally sensitive mental health care is necessary to lead those children in need of help to mental health care. This should be an important topic in future research.

### Strengths and limitations

To our knowledge, this is the first study that investigated characteristics of a high-risk ethnic subgroup of childhood offenders. We were able to use official police registrations for offending and re-offending. In addition to these official police registrations concerning the child, we also had access to police data of household members of the child. Furthermore, we gathered information from parent and child reports and information from the Child Welfare Agency.

Our study has several limitations. First, while officially registered offending is in particular a strong predictor for a persistent pattern of delinquency, it has also some disadvantages: Since there is no penal code for children below the age of twelve it remains unknown whether the registered child is actually guilty. In addition, only a part of delinquent behaviour is actually registered by the police; there is no information on the dark number. Second, additional information from teachers would have been helpful to clarify the results on psychosocial functioning and would have helped to interpret the socially desirable answers. Although we tried to make use of teacher reports, the response rate was too low to be useful. Possibly because of the controversy of the topic, the parents were reluctant to give permission to contact teachers. Third, although we followed up on police data, we did not follow up on the parent and child reports. Therefore we were not able to take the child's development into account. Longitudinal data would have provided information on characteristics of those who continue to offend and would also have yielded important information on characteristics of desistance of childhood offending.

Despite these limitations, this study has helped us gain insight into characteristics of offending and re-offending in a high-risk subgroup of childhood offenders. This information is needed to develop future interventions that contribute to a healthy development for these vulnerable boys.

## Competing interests

The authors declare that they have no competing interests.

## Atuhors' contributions

CP carried out the study. LvD participated in its design and coordination and helped to draft the manuscript. GS helped to draft the manuscript. TD conceived of the study and revised the manuscript critically. All authors have read and approved the final manuscript.

## Endnotes

^1 ^Childhood delinquency and offending refer to behaviour that can be prosecuted if the person has reached the age of criminal responsibility. In the Netherlands, the age of criminal responsibility is twelve years. In this paper, children are also called delinquent if not taken to the police station, but only reprimanded on the street. Substance use and status offences such as running away and truancy have been excluded.

^2 ^In general, over one quarter of the children in Amsterdam finish elementary school with a reading deficit [81], compared to 41.4% to 81.3% of the boys in our study. Furthermore, while in the general population about 2% repeat a class in the Netherlands in this age group [82], in our study 16.4% to 37.5% repeated a class.

^3 ^For comparison, in the general Moroccan population in the Netherlands, about 15% of the households are single-parent households [83]. In our study 33.8% of one-time offenders and 22.9% of re-offenders lived with a single parent. With about one quarter of Moroccan families in the Netherlands living below the poverty line, financial problems are relatively common in this group [84]. However, in our study the majority of parents said they had financial problems. Furthermore, in the general population about 10 percent of Moroccan juveniles are registered as suspects by the police [25], while in our study over one quarter to two thirds of the boys lived with an arrested family member. Finally, Dutch national figures of police registered domestic violence are around 12% compared to over 35% in our study across all groups [85].
